# Remote Vital Sign Monitoring in Acutely Unwell Hospital at Home Patients: Nonrandomized Feasibility Study

**DOI:** 10.2196/84233

**Published:** 2026-09-25

**Authors:** Rachel C Greer, Cristian Roman, Mayue Shi, Benn Gooch, Bethany K Lawson, Christopher Biggs, Clare Edwards, Sarah Vollam, Peter Watkinson, Lionel Tarassenko, Andrew Farmer, Daniel Lasserson

**Affiliations:** 1 Nuffield Department of Primary Care Health Sciences University of Oxford Oxford, England United Kingdom; 2 NIHR Oxford Biomedical Research Centre Oxford, England United Kingdom; 3 Department of Engineering Science Institute of Biomedical Engineering University of Oxford Oxford, England United Kingdom; 4 Research Centre for Biomedical Engineering School of Science and Technology City St George's, University of London London, England United Kingdom; 5 Nuffield Department of Clinical Neurosciences University of Oxford Oxford, England United Kingdom; 6 Department of Geratology, Acute Hospital at Home Oxford University Hospitals NHS Foundation Trust Oxford, England United Kingdom; 7 Warwick Medical School, Warwick Applied Health University of Warwick Coventry, England United Kingdom

**Keywords:** remote monitoring, vital signs, hospital at home, virtual wards, wearables

## Abstract

**Background:**

Remote vital sign monitoring of acute hospital at home (aHAH) patients is recommended in policy without clear guidance on implementation. While such monitoring has the potential to improve patient care, there is limited evidence for its feasibility and acceptability.

**Objective:**

This study aimed to evaluate the feasibility and acceptability of using a remote, community-based monitoring system using a vital sign wearable patch and pulse oximeter in aHAH patients.

**Methods:**

In this nonrandomized feasibility study, we recruited patients from an aHAH service in Oxford, United Kingdom, between January and October 2024. Eligible patients were aged 18 years and older with an acute illness (lower respiratory tract infection, cellulitis, urinary tract infection, systemic evidence of acute infection, acute kidney injury, or heart failure with acute fluid overload) requiring aHAH care. Pregnant women and those with contraindications to monitoring were excluded. Participants were asked to wear a chest patch to estimate their heart rate and respiratory rate (passive monitoring), and to intermittently check their oxygen saturations, blood pressure, and temperature (active monitoring). Participants were asked to complete a Technology Acceptance Questionnaire. All aHAH patients were screened. Recruitment was nonconsecutive due to the availability of the research team. Participants were monitored for 7 days or until discharge from the aHAH service, whichever was shorter. Fixed time windows were used to assess real-time vital sign data coverage during each participant’s monitoring period. The primary outcomes were the proportion of 4-hour monitoring windows with a recorded heart rate and 12-hour daytime windows with a recorded oxygen saturation level. Time-series analyses and descriptive statistics were used for quantitative data. Content analysis was used to analyze the open-ended comments in the questionnaire.

**Results:**

In total, 29 participants were recruited and 3 immediately withdrew from the study. Participants were monitored for 4.8 (IQR 2.9-6.2) days on average. Overall, 89% (600/674) of the 4-hour monitoring windows had a heart rate and 75.4% (508/674) had a respiratory rate recorded in real-time. For the 12-hour daytime windows, 58.1% (75/129) had an oxygen saturation, 54.3% (70/129) had a blood pressure, and 51.2% (66/129) had a temperature recorded. Data coverage was higher for passive monitoring compared to active monitoring. Most participants who had capacity found the combined monitoring system easy to use and thought it was useful for their health care.

**Conclusions:**

Remote monitoring of vital signs in aHAH patients is feasible. Good data coverage was achieved for the passive monitoring, which did not require specific actions by the participants or their caregivers. Further work is required to ascertain which patients would benefit most from this monitoring.

## Introduction

Remote vital sign monitoring has the potential to improve clinical care and efficiency within acute hospital-at-home (aHAH) services and is mandated in policy, yet little is known about its feasibility and acceptability among patients. aHAH services are being established across the National Health Service (NHS) in the United Kingdom to provide hospital-level care to patients’ homes; this is in part due to the recognition that hospital-based care has associated risks, especially for people living with frailty [1,2]. aHAH can replace the need for hospital admission and support early discharge from hospital, reducing the risk of hospital-acquired infections, delirium, and physical deconditioning, relieving pressure on hospital beds, and moving care away from the hospital to the community [1,3,4]. However, the contribution of remote monitoring technologies in this setting to supporting clinical decision-making and contributing to patient safety remains unclear.

Remote monitoring of acutely unwell patients at home has the potential to allow the treatment and care of patients to be modified and made more efficient, for example, by prioritizing clinician visits and escalating or deescalating treatment. Measures of vital signs are a key component of early warning systems to identify patients at risk of deterioration [5]. However, guidance is lacking as to which vital signs should be monitored in aHAH settings, how frequently, and by which method [6].

Vital sign monitoring can involve continuous or intermittent collection of data; it may be active, relying on the patient or their caregiver taking, recording, and reporting the readings, or passive, relying on automated recording and transmission of wearable data to the clinician [7]. There is a lack of data on remote vital sign monitoring in the community [8,9]. A recent systematic review of remote monitoring in trials comparing aHAH and inpatient hospital care only identified two trials describing active vital sign monitoring and two studies describing passive monitoring using a chest patch. No trials were identified comparing active or passive monitoring, and the reliability or feasibility of monitoring was not discussed [8,10-13]. A rapid review of remote monitoring for patients with COVID-19 found that most studies monitored oxygen levels and were dependent on the patient reporting these levels to the clinicians, while only a minority of studies used direct reporting from the devices [14].

Current aHAH practice often involves measurement of vital signs when a clinician visits the patient. While use of remote monitoring technologies is recommended in policy with the aim of encouraging innovation and wider adoption, evidence for the use and interpretation of the technology is lacking, as are guidelines for the implementation of remote monitoring [15-18]. The uptake of remote monitoring in standard care has been limited and procured equipment remains unused [17,19,20]. Previous research has identified the importance of embedding and refining technological solutions in clinical care at an early stage of their development [16].

In this study, we aimed to pilot the use of a remote, community-based monitoring system using a wearable vital sign patch and pulse oximeter in aHAH patients. We also explored the remote monitoring of blood pressure (BP) and temperature in this patient group. Here, we report on the feasibility and acceptability of this remote vital sign monitoring system.

## Methods

### Overview

We conducted a nonrandomized feasibility study, enrolling patients with an acute illness who were being cared for by Oxford University Hospitals’ aHAH team.

### Study Setting

Oxford University Hospitals’ central aHAH team, based at the John Radcliffe Hospital in Oxford, United Kingdom, is led by a medical consultant and consists of a multidisciplinary team including doctors, nurses, allied health professionals, and pharmacists. It aims to provide hospital-level care in patients’ homes, allowing them to avoid hospital admission or shorten their hospital stay [21]. The care model within the aHAH is designed to replace acute hospital admission, with daily face-to-face visits for medication delivery and clinical assessment, and when there is de-escalation after the acute phase of treatment, there can be a step-down phase where the visiting frequency may be decreased to ensure clinical stability before discharge back to primary care. The majority of referrals to the aHAH service are intended to avoid hospital admission (hospital avoidance) and are received directly from community services including the patient’s general practice team. Referrals are also received from the hospital, including its Ambulatory Assessment Unit and A&E department. Referrals are triaged by a senior decision maker (consultant doctor or senior nurse). The initial aHAH assessment is usually carried out in the patient’s home but can also be undertaken in the hospital depending on the patient’s location at the time of referral and the urgency of the referral. Medical consultants oversee the patients’ care throughout their aHAH admission. The aHAH team is equipped to carry out point-of-care investigations such as blood tests and ultrasound scans in the patient’s home. Treatments include oxygen, nebulizers, intravenous medicines and fluids, and end-of-life care. It is staffed from 8 AM to 8 PM, 7 days a week, with overnight support from existing emergency services with access to the patients’ electronic health records. Prior to the establishment of this service, patients would have been admitted to hospital for investigation and treatment.

### Recruitment

Eligible patients for the study were aged 18 years or older and appropriate for home management of an acute illness requiring hospital-level diagnostic tests and treatment. They were initially diagnosed with at least one of the following: lower respiratory tract infection, cellulitis, urinary tract infection, systemic evidence of acute infection, acute kidney injury, or heart failure with acute fluid overload. Participants needed to be able to wear a vital sign monitoring chest patch for up to 7 days and to give their informed consent (or consultee declaration) to take part in the study. Exclusion criteria included pregnancy, inability to comply with the study procedures, and contraindications to monitoring. The research team needed to be satisfied that remote monitoring could be safely established at the patient’s home, which included the need to have sufficient mobile phone coverage for data transfer.

Potential participants were identified directly from the aHAH team or by screening their patient lists. Eligibility assessments were conducted on all aHAH patients using the patient’s medical records. Recruitment was nonconsecutive and took place during office hours, Monday to Friday, when research staff were available; potentially eligible patients were approached by the research team, and enrolment took place as soon as possible after patients were referred to the aHAH service. Participants were recruited in their own homes or at the hospital’s Ambulatory Assessment Unit if they were being assessed prior to return home. If eligible and willing to join the study, participants were asked to provide their written or verbal informed consent to participate by a study clinician prior to undertaking initial study procedures.

### Study Procedures

Participants were asked to wear a monitoring system consisting of the vital sign monitoring chest patch (passive monitoring) and to wear a pulse oximeter at regular intervals for a short period of time (active monitoring). Participants were also asked to check their BP and temperature as often as they were able and record them in a paper-based diary (active monitoring). Participants were monitored until their discharge from the aHAH team or for a maximum of 7 days, whichever was shorter. Sociodemographic details were collected from the participants at recruitment. Clinical characteristics were gathered from the medical records.

Participants received usual standard care and monitoring from the aHAH team throughout the study period. Data from the remote monitoring system were available to the aHAH in real-time, and data were reviewed daily. Alerts were not added to the monitoring system. Participants were asked to contact the aHAH team if they were concerned or feeling more unwell, following the usual treatment pathway. Formal analysis of the remote monitoring data was conducted retrospectively.

Participants were asked to complete a Technology Acceptance Questionnaire over the phone within 4 weeks of completing the study monitoring period (Multimedia Appendix 1). The questionnaire was contextually adapted from the Technology Acceptance Model and consists of six statements relating to the ease of using the monitoring devices and whether they were useful for participants’ health care [22]. The questions were modified to reflect the specific technology and care setting under investigation while maintaining the original construct domains. Participants were asked to rank their agreement with the statement using a 7-point Likert scale [23]. Open-ended comments were recorded for each of the six statements and alongside any overarching additional comments.

### Ethical Considerations

Ethical approval was received from the London - Camden & Kings Cross Research Ethics Committee, reference 23/LO/0559, and the Health Research Authority, IRAS project ID 325766. Section 251 approval was given by the Confidentiality Advisory Group to permit direct screening of patient medical records to assess the suitability of potential participants. All participants provided written or verbal informed consent to participate. If participants lacked capacity to consent to participate in research, then consultee declarations were used. These participants were not asked to complete the Technology Acceptance Questionnaire. Participants’ vital sign data were pseudo-anonymized on the clinical dashboard using their hospital numbers, allowing linkage to their medical records by the aHAH team. The clinical dashboard was hosted on NHS servers and password-protected. Study-IDs were used to pseudo-anonymize the other research data. No compensation was provided to the participants.

### Monitoring System

The remote monitoring system was developed by our research team using commercially available clinical-grade wearables and was evaluated for accuracy and wearability in healthy volunteers and hospital inpatients [24,25]. It consists of the VitalPatch (VitalConnect) chest patch [26] and WristOx 3150 OEM Bluetooth-Low Energy (Nonin Medical Inc) pulse oximeter [27]. The VitalPatch records a single-lead electrocardiogram (ECG), electrical impedance pneumography (EIP), and 3-axis accelerometer waveforms. A heart rate, respiratory rate, patient posture (eg, standing, sitting, and lying down), and step count are derived from the ECG, EIP, and accelerometer waveforms by the VitalPatch. The VitalPatch battery lasts for 7 days. The pulse oximeter measures the pulse rate, peripheral oxygen saturation, and records a near-infrared photoplethysmogram waveform.

In our monitoring system, data from both wearable devices are collected on a tablet computer (Samsung Tab A 2019, SM-T585) and then transmitted over mobile data networks to a remote Oxford University Hospitals NHS server, where the data can be viewed live using the clinical dashboard (Figure 1).

To remove motion artefact as much as possible, the real-time transmitted vital-sign data stream was summarized using the median estimator for each 30-second window. To allow large periods of live vital-sign data to be browsed retrospectively from the clinician dashboard, the research team decided to limit the resolution of the live data to 5-minute windows. Five-minute median values of heart rate, respiratory rate, and oxygen saturation were presented in a graph alongside the most recent number of steps and patient posture.

In addition to the monitoring system, participants were supplied with a rigid cuff arm BP monitor (Omron Evolv) [28] and a tympanic thermometer (Fora IR20b, ForaCare) [29].

**Figure 1:**
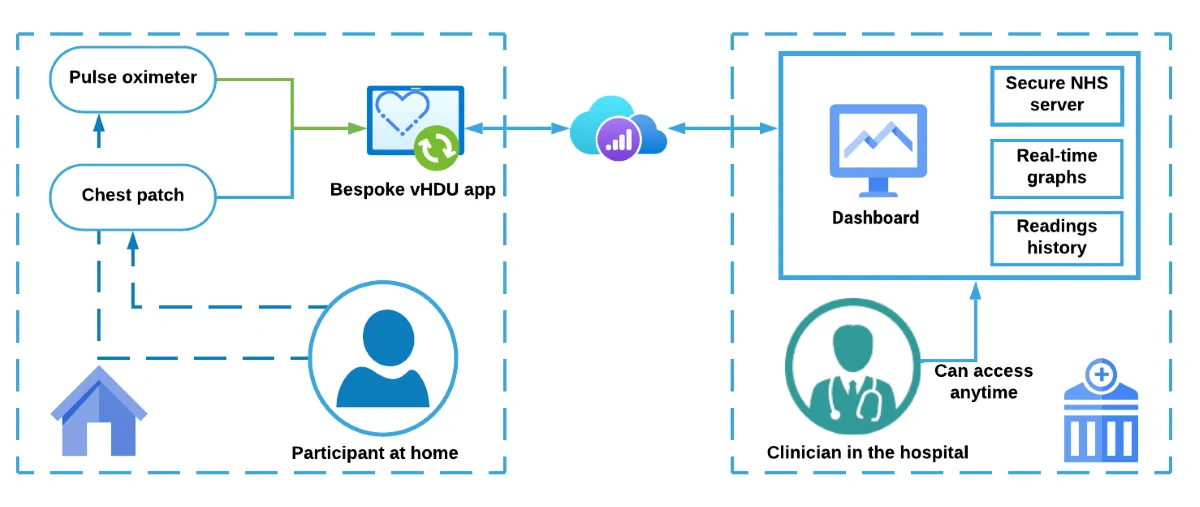
Diagram of the transfer of data from the remote monitoring system. NHS: National Health Service; vHDU: virtual high dependency unit.

### Outcome Measures

The primary outcome was the proportion of 4-hour monitoring windows with a recorded heart rate and the proportion of 12-hour daytime windows with a recorded oxygen saturation level during the study period.

Secondary outcomes were the proportion of 1-hour monitoring windows with a recorded heart rate, 24-hour windows with a recorded oxygen saturation, 1-hour and 4-hour windows with a recorded respiratory rate, and the feasibility of intermittent BP and temperature monitoring using 12-hour daytime and 24-hour windows. The time windows were chosen in line with the recommended monitoring windows for NHS hospitals, which are based on the National Early Warning Score 2 (NEWS2). This recommends a minimum of 12 hourly monitoring for all patients (NEWS2=0), 4 to 6 hourly for those at low risk (NEWS2=1-4), and hourly for those at moderate risk (NEWS2=5-6 or 3 in a single parameter) [30]. The 12-hour daytime window for active monitoring (oxygen saturation, BP, and temperature) was chosen to be 8 AM to 8 PM to coincide with the core working hours of the aHAH team. The study protocol specified thresholds between 60% and 100% for data coverage as the study outcome.

The acceptability of remote vital sign monitoring is reported narratively using quantitative data and comments made during the Technology Acceptance Questionnaire.

Clinical outcomes, including re-admission to aHAH, admission to hospital, and death, were collected from the medical records 6 months after enrolment.

### Statistical Analysis

Data were summarized using descriptive statistics and time-series analyses. Medians and histograms were used for quantification and visual representation of the primary outcome. Vital sign data coverage was analyzed using fixed time windows and the number of windows with and without data were calculated. The time windows were aligned to fixed clock times within each calendar day. For example, the 4-hour windows started uniformly at midnight, 4 AM, and so on.

The first time window reported for each participant corresponds to the first received heart rate value (eg, if the first reading is received at 11:30 AM, then the first window is 8 AM-noon). The last window corresponds to the last received heart rate value, discharge from aHAH, or 7 days of monitoring, whichever is shortest.

Inductive content analysis was used to analyze the open-ended comments in the Technology Acceptance Questionnaire. Units of meaning were identified in the data and subsequently coded and grouped into categories by one reviewer (RCG). The codes and categories were discussed with another reviewer (AF) to ensure consensus. The final content is described [31].

Analyses were conducted using Python (version 3.11.5) and STATA 18 (College Station, TX). Data visualization was performed using Matplotlib (version 3.8.2). Missing vital sign data were left as gaps in the time series and were not imputed.

### Sample Size

A sample size of 35 was prespecified to provide a range of participants in whom to assess the feasibility and acceptability of remote vital sign monitoring. The sample size determination was made on the basis of guidance for feasibility and pilot studies [32,33].

### Patient and Public Involvement

Patient and public representatives’ opinions were sought from the research team’s established Patient and Public Involvement and Engagement Group on the value of remote monitoring and the study design, including acceptability of screening using medical records prior to consent and enrolling participants without capacity. The monitoring system was also demonstrated and discussed with members of the public at a research showcase public event.

## Results

### Overview

In total, 54.5% (214/393) aHAH patients were potentially eligible to join the study. Of these, 52 (24.3%) were approached, and 29 participants were enrolled in the study between January and October 2024 (Figure 2). Of these, 3 immediately withdrew (2 were unable to wear the chest patch and 1 withdrew their consent) and were not considered further. The main source of referrals to the aHAH service changed during study preparation and recruitment, with increasing numbers of patients being referred and assessed directly in the community without attending the hospital. We therefore paused recruitment between February and July 2024 to amend the protocol to allow for recruitment in participants’ homes. The final participant finished their monitoring period at the end of October 2024. Recruitment ceased when the investigators determined post hoc that the accumulated data were sufficient to address the study objectives and that further participants would not change the conclusion about the extent of data completeness.

The median age of the participants was 77 years, 15.4% (4/26) lived in a care or residential home, and 42.3% (11/26) lacked capacity to consent to participate in the study and were enrolled with a consultee declaration (Table 1). The most frequent clinical presentations were breathlessness (13/26, 50%) and/or limb swelling (8/26, 30.8%). Almost half were initially diagnosed with a lower respiratory tract infection (12/26, 46.2%) and/or heart failure (11/26, 42.3%). The median study monitoring period was 4.8 (2.9-6.2) days. The median length of aHAH admission was 9.9 (3-15.1) days; this was longer than the study period if participants were admitted for more than 7 days. The outcomes at discharge from the initial aHAH admission (where the study monitoring took place) were discharge (21/26, 80.8%), admission to hospital (4/26, 15.4%), and death (1/26, 3.9%). Of the 4 participants who were admitted as inpatients, 2 admissions took place during the study monitoring. Their vital signs during their admission in the aHAH were abnormal prior to admission to hospital. The participant who died while receiving care from the aHAH service had finished their period of study monitoring. No deaths or admissions were judged to be related to the study. Within 6 months of study enrolment, 36% (9/25) had a subsequent admission to the aHAH service and 56% (14/25) had been admitted to hospital as an inpatient.

**Figure 2:**
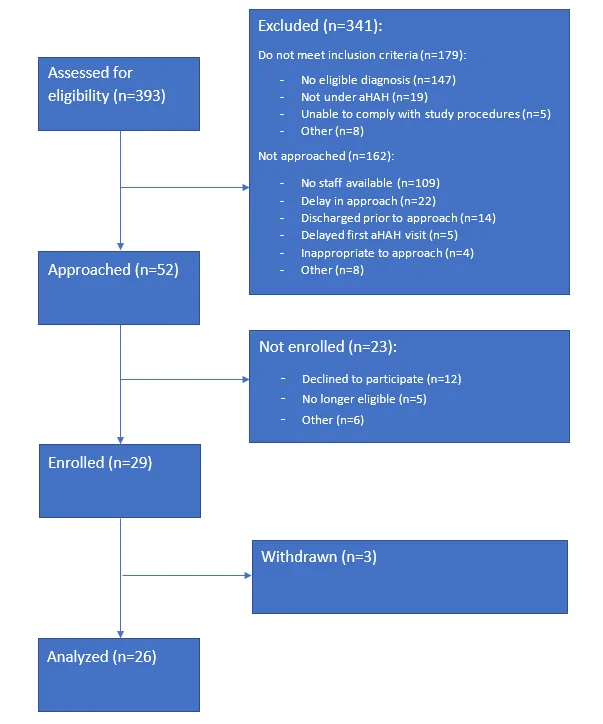
Participant flow diagram.

**Table 1:** Demographic and clinical characteristics of the participants.

Characteristics		Number of participants
Age (years), median (IQR)		77 (67-86)
Sex male (N=26), n (%)		12 (46.2)
Ethnicity (N=26), n (%)		
	British^a^	24 (92.3)
	Any other White background	1 (3.9)
	White and Black Caribbean	1 (3.9)
Living arrangements (N=26), n (%)		
	Own home	22 (84.6)
	Care or residential home	4 (15.4)
Do you live on a single level? (N=26), n (%)		
	Yes	9 (34.6)
	No	13 (50)
	Care or residential home	4 (15.4)
Who do you live with? (N=26), n (%)		
	Live alone	6 (23.1)
	With spouse	12 (46.2)
	With another family member	3 (11.5)
	With others	1 (3.9)
	Care or residential home	4 (15.4)
Care package in place (N=26), n (%)		
	Yes	9 (34.6)
	No	17 (65.4)
Frequency of carer visits (N=9), n (%)		
	Twice a day	3 (33.3)
	4 times a day	1 (11.1)
	Continuous care (≥5 times a day)	5 (55.6)
Clinical presentation^b^ (N=26), n (%)		
	Breathlessness	13 (50)
	Limb swelling	8 (30.8)
	Acute functional decline	6 (23.1)
	Fever	5 (19.2)
	Confusion or disorientation	3 (11.5)
	Pain	2 (7.7)
	Delirium	1 (3.9)
	Fall	1 (3.9)
	None of the above	3 (11.5)
Initial diagnosis^b^ (N=26), n (%)		
	Lower respiratory tract infection	12 (46.2)
	Heart failure with fluid overload	11 (42.3)
	Urinary tract infection	4 (15.4)
	Acute kidney injury	2 (7.7)
	Cellulitis	1 (3.9)
	None of the above	2 (7.7)
Past medical history^b^ (N=26), n (%)		
	Heart failure	12 (46.2)
	Chronic kidney disease	9 (34.6)
	Diabetes	6 (23.1)
	Chronic obstructive pulmonary disease	5 (19.2)
	Cancer	5 (19.2)
	Dementia	4 (15.4)
	Obesity	2 (7.7)
	None of the above	3 (11.5)
Rockwood Clinical Frailty Score, median (IQR), (N=19)^c^		6 (4-7)
NEWS2^d^ score closest to the referral to aHAH^e^, median (IQR)		2.5 (1-4)
Lacked capacity to consent to research (N=26)		11 (42.2)
Treatment before referral to aHAH (N=26), n (%)		
	Intravenous antibiotics	5 (19.2)
	Intravenous diuretics	3 (11.5)
	Oxygen	1 (3.9)
	Intravenous fluids	1 (3.9)
Planned treatment after referral to aHAH (N=26), n (%)		
	Intravenous antibiotics	6 (23.1)
	Intravenous diuretics	7 (26.9)
	Oxygen	0 (0)
	Intravenous fluids	1 (3.9)
Length of study monitoring (days), median (IQR), maximum 7 days)		4.8 (2.9-6.2)
Length of aHAH admission (days), median (IQR)		9.9 (3-15.1)
aHAH visits per day of monitoring, median (IQR)		0.8 (0.5-1)
During the monitoring period at least one (N=26), n (%)		
	Point-of-care blood test	19 (73.1)
	Intravenous diuretic	8 (30.8)
	Intravenous antibiotic	7 (26.9)
	Intravenous fluids	3 (11.5)
	Point-of-care ultrasound	3 (11.5)
	Phone review	2 (7.7)
Outcome of the initial aHAH admission^f,^ n (%)		
	Discharged (N=26)	21 (80.8)
	Palliative (N=21)	4 (19)
	Admitted to hospital (N=26)	4 (15.4)
	Death (N=26)	1 (3.9)
Readmitted under aHAH (N=25), n (%)		
	Within 30 days of initial aHAH discharge	8 (32)
	Within 6 months of study enrolment	9 (36)
Admitted to hospital (N=25), n (%)		
	Within 30 days of initial aHAH discharge	9 (36)
	Within 6 months of study enrolment	14 (56)
Death (N=26), n (%)		
	Within 30 days of initial aHAH discharge	4 (15.4)
	Within 6 months of study enrolment	8 (30.8)

^a^British: English, Welsh, Scottish, North Irish or British.

^b^Can have more than one response.

^c^Rockwood Frailty Score: 1=very fit, 2=well, 3=managing well, 4=vulnerable, 5=mildly frail, 6=moderately frail, 7=severely frail, 8=very severely frail, 9=terminally ill.

^d^NEWS2: National Early Warning Score 2.

^e^aHAH: acute hospital at home.

^f^The initial aHAH admission is defined as the aHAH admission during which the study monitoring took place.

### Real-Time Remote Monitoring Data

All participants had a heart rate and respiratory rate recorded and reported in real-time to the clinical dashboard during the study. All but 1 participant (25/26, 96.2%) had an oxygen saturation level recorded. The pulse oximeter was not linked to the monitoring system for the missing participant. One participant was monitored for less than 4 hours, so is not considered further. In total, 25 participants were monitored for 674 x 4-hour windows, in which 600 (89%) had a heart rate recorded. When broken down into 1-hour windows, 81.3% (2114/2599) had a heart rate recorded. Data missingness for passive monitoring (VitalPatch heart rate values) was not concentrated at particular times of day, prolonged gaps (>4 hours) were uncommon (median of 1 per participant), and more active participants had more missing data, consistent with temporary tablet out-of-range disconnections (Multimedia Appendix 2). Oxygen saturations were recorded in 58.1% (75/129) of the 12-hour daytime windows and 61.2% (79/129) of the 24-hour windows. A respiratory rate was recorded in 69.4% (1803/2599) of the 1-hour and 75.4% (508/674) of the 4-hour windows (Table 2). Additional vital sign data were recovered from the chest patch and tablet retrospectively when connectivity between the VitalPatch and tablet was restored but are not displayed in these figures, which represent only the data available in real-time.

Examples of data coverage for 4 participants are shown in Figure 3.

**Table 2:** The proportion of time windows with a real-time recorded vital sign (heart rate, oxygen saturation, respiratory rate, blood pressure, and temperature).

Vital sign			Proportion of windows with a recorded vital sign, n/N (%)
Heart rate			
	1-hour window	2114/2599 (81.3)	
	4-hour window	600/674 (89)	
Oxygen saturations			
	12-hour window (8 AM-8 PM)	75/129 (58.1)	
	12-hour window (8 PM-8 AM)^a^	30/111 (27)	
	24-hour window (8 AM-8 AM)	79/129 (61.2)	
Respiratory rate			
	1-hour window	1803/2599 (69.4)	
	4-hour window	508/674 (75.4)	
Blood pressure			
	12-hour window (8 AM-8 PM)	70/129 (54.3)	
	12-hour window (8 PM-8 AM)^a^	33/111 (29.7)	
	24-hour window (8 AM-8 AM)	84/129 (65.1)	
Temperature			
	12-hour window (8 AM-8 PM)	66/129 (51.2)	
	12-hour window (8 PM-8 AM)^a^	27/111 (24.3)	
	24-hour window (8 AM-8 AM)	77/129 (59.7)	

^a^Participants may have fewer nocturnal (8 PM-8 AM) 12-hour monitoring windows as they were recruited into the study and discharged from aHAH during daytime hours.

**Figure 3:**
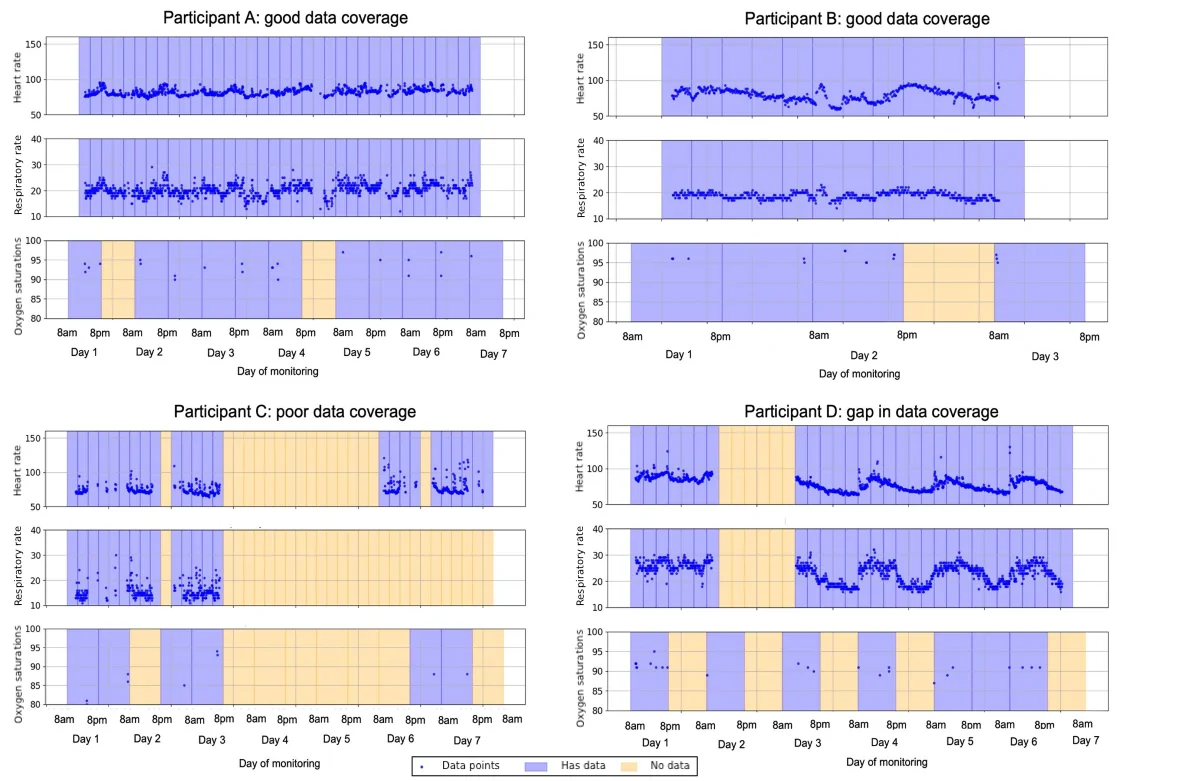
Examples of individual data coverage for 4 participants.

### Participant Diaries

Participant diaries detailing the BP and temperature readings were returned for all but 1 participant. Of these, 83.3% (20/24) recorded at least one BP reading and 75% (18/24) recorded at least one temperature reading; 1 participant commented that they could not get the thermometer to work. Family members and caregivers helped some participants complete their participant diaries. At least one BP and one temperature reading were recorded for 80% (8/10) and for 60% (6/10) of participants who lacked capacity, respectively. In total, participants were monitored for 129 x 12-hour daytime windows, 70 (54.3%) of which recorded a BP reading and 66 (51.2%) recorded a temperature reading.

### Technology Acceptance Questionnaire

The Technology Acceptance Questionnaire was answered by 38.5% (10/26) of participants. Participants who lacked the capacity to join the research were not approached to complete the questionnaire (11/26, 42.3%). Other reasons for not completing the questionnaire included loss to follow-up, questionnaire declined, and end-of-life care (5/26, 19.2%).

The responses were mostly positive, with 80% (8/10) agreeing that the monitoring devices were easy to use and useful for their health care. A few participants lacked clarity about what to do with the monitoring devices (Table 3).

Additional comments made by participants relating to challenges with the monitoring devices included noting that the thermometers were hard to use (n=2), difficulty using the BP monitors or confusion over which numbers to record (n=2), and accuracy of the pulse oximeter (n=1). Factors influencing ease of use included the devices, being unwell, previous experiences, ways of learning, and the explanations received. Two participants said family members helped to take or record readings. When asked if they felt the monitoring devices were useful for their health care, one participant said they felt safer at home, another was positive about being monitored, while another was unsure due to limited feedback.

**Table 3:** Technology Acceptance Questionnaire responses.

Statements	Ratings^a^, n (%)						
	1	2	3	4	5	6	7
Using the monitoring devices while I was unwell was easy for me	0 (0)	0 (0)	0 (0)	0 (0)	1 (10)	2 (20)	7 (70)
It was easy to get the monitoring devices to do what I wanted them to do	0 (0)	0 (0)	0 (0)	0 (0)	0 (0)	2 (20)	8 (80)
What I needed to do with the monitoring devices was clear and Understandable	0 (0)	0 (0)	1 (10)	1 (10)	1 (10)	2 (20)	5 (50)
It was easy for me to learn what I need to do with the monitoring devices	0 (0)	0 (0)	2 (20)	0 (0)	0 (0)	1 (10)	7 (70)
I found the monitoring devices easy to use	0 (0)	0 (0)	0 (0)	0 (0)	1 (10)	1 (10)	8 (80)
I felt that having the monitoring devices was useful for my health care	0 (0)	0 (0)	0 (0)	2 (20)	0 (0)	0 (0)	8 (80)

^a^1=completely disagree, 2=mostly disagree, 3=somewhat disagree, 4=neutral, 5=somewhat agree, 6=mostly agree, 7=completely agree.

## Discussion

### Principal Findings

Remote vital sign monitoring is feasible in patients being cared for by aHAH services. Even though our population was older and included patients living with frailty and multimorbidity, including cognitive impairment, remote vital sign data were recorded and transferred to a central server in the NHS organization providing aHAH care. However, there was a difference between active and passive monitoring.

Passive monitoring of participants’ heart rates and respiratory rates was feasible in the majority of participants, with almost 90% and 75% respectively having live data in each 4-hour window of monitoring. Prolonged gaps in missing heart rate data were uncommon, consistent with findings from feasibility studies of remote vital sign monitoring in patients following surgery [34,35]. The positive association between patients’ step counts and missing heart rate data may reflect temporary interruptions in data transmission when the tablet was out of range. Alternative devices for data storage and transmission, such as mobile phones or smartwatches, may help to address this limitation. Active monitoring of vital signs by participants (oxygen saturations, BP, and temperature) was less reliable and fell below our prespecified threshold of 60% data coverage.

Comparisons of passive and active remote vital sign monitoring are lacking in the literature [8]. In line with our results, a feasibility study monitoring multiple sclerosis symptoms found adherence to passive monitoring was higher than that to active monitoring [36]. Levels of active monitoring may be reduced by the burden of monitoring and technical challenges. In our study, the burden of active monitoring may have been increased because of the participants’ acute illness or preexisting frailty, resulting in some participants needing support from family members and carers. Participants were asked to manually record their BP and temperature in handwritten diaries, which added an additional step to the monitoring and may have reduced adherence. Oxygen saturation levels were automatically recorded by the monitoring system but still required the participants to wear the pulse oximeter. Previous studies have highlighted the burden of active monitoring on patients and their caregivers and identified this as a potential barrier to implementation [17]. Technical challenges can also present implementation challenges, including the usability of devices and the digital skills required to conduct remote monitoring [37]. This is problematic where active remote monitoring is considered essential for patients’ clinical management.

This study adds detailed evidence for the feasibility and reliability of remote vital sign monitoring of frail, acutely unwell patients, an area in which evidence is lacking in the literature. The majority of our participants found the monitoring devices easy to use when unwell, which is particularly important in a group who would otherwise require admission to hospital. Carers and family members helped some participants with actively monitoring their BP and temperature. Overall, our participants reported that the monitoring devices were useful for their health care and some reported feeling safer at home with the monitoring. This is supported by other studies in which monitoring gave patients peace of mind and provided reassurance [38-40]. Studies have reported that patients can feel concerned or anxious about their abnormal readings, although some patients went on to say they were reassured that their clinicians would be notified and act on the findings [17,38,40].

### Clinical Implications

Remote monitoring can only provide a safety net to those being cared for at home if the monitoring is achievable, the data are reliable, and appropriate systems are in place for clinicians to respond to any deterioration.

Passive vital sign monitoring is less reliant on the patient or their carer, so it could particularly benefit frail, acutely unwell patients and those who are less comfortable with technology. There is growing awareness of the need to prevent digital exclusion. The passive nature of monitoring with the chest patch reduces this risk of digital exclusion. Any system reliant on active remote monitoring would need to promote inclusion to ensure that a 2-tier system was not created, with enhanced care being offered to those digitally able while leaving those less able behind, thereby widening health inequalities. Remote monitoring has the potential to support the management of patients in more rural locations where distances from the hospital or center of care may be a barrier to care.

Remote monitoring has the potential to make aHAH teams more efficient by directing resources (including the seniority of clinical staff and frequency of visits) toward those most in need. It could also help to quantify the level of risk and clinical acuity of patients on a continuous basis rather than relying on in-person assessments to provide episodic insight. Although we were not calculating a full NEWS2 score on all component variables remotely, the heart rate and respiratory rate data could be used to update patients’ level of clinical risk more frequently than current recommendations for low-risk patients being cared for in hospital (at least 4-hourly), in addition to the in-person assessments conducted by aHAH teams [30]. These insights could be used to step up or down treatment and detect clinical deterioration earlier. Prior studies have considered the impact of home remote monitoring in patients following hospital discharge after major surgery; while the monitoring was feasible, it did not affect the clinical care of the patients [34,35].

Further work is needed to address key questions including which patients require remote monitoring, whether passive monitoring is sufficient in all cases, and how the monitoring should best be incorporated into clinical care pathways [16,39]. The impact of remote monitoring on patient and health system outcomes, including clinical outcomes, workloads, and cost-effectiveness, also needs to be evaluated [6,8,14,39]. Appropriate alert thresholds for vital sign monitoring in aHAH settings need to be developed rather than relying on hospital-based scores, enabling detection of clinical deterioration without falsely alarming patients or clinicians and unnecessarily increasing workloads. Raw data from continuous monitoring need to be summarized to aid interpretation by clinicians and reduce false alerts from short-term fluctuations in vital sign measurements. Trends in the trajectories of individual patients’ vital signs may be more informative than intermittent calculations of vital sign warning scores such as NEWS2. Short gaps in data coverage may therefore be tolerated as long as the trend in the trajectory is maintained. Adequate data coverage will depend on the clinical situation, the patient’s acuity and how remote monitoring is incorporated into clinical care; this requires further exploration.

Alongside the clinical need and impact of remote monitoring of aHAH patients, the scalability of remote monitoring also needs to be considered. To be scalable, monitoring systems need to be interoperable and integrated with existing electronic health records [16,17,24,41]. As with all digital health interventions, training for patients and clinicians and technological support are necessary to facilitate the introduction of remote passive monitoring into clinical care [14,16,42].

The study does have some limitations: on the one hand, participant feedback from the Technology Acceptance Questionnaire has helped us to understand the acceptability of remote monitoring in this patient group, although a more nuanced and detailed understanding of acceptability would require in-depth qualitative methods. We did not ask those who lacked capacity or their carers to complete the Technology Acceptance Questionnaire. However, the inclusion of participants who lacked capacity and those living with frailty in the main study is a strength and reflects the medical complexity of patients who may benefit the most from remote monitoring. aHAH services and the populations they serve are varied. Recruitment into the study was from a single Trust’s aHAH service and was nonconsecutive, which may limit the generalizability of these results. Nevertheless, the majority of aHAH services fall into respiratory, heart failure, or frailty categories, which are all represented in our diverse patient population. The main source of referrals to the aHAH service changed during study preparation and recruitment, with increasing numbers of patients being referred and assessed directly in the community without attending the hospital. We therefore paused recruitment between February and July 2024 to amend the protocol to allow for recruitment in participants’ homes.

### Conclusions

Our findings demonstrate the feasibility of remotely monitoring vital signs in aHAH patients. Additional research is required to ascertain whether passive monitoring is sufficient, and if not, further feasibility work is needed to support patients in active monitoring. Further work is needed to identify when and how remote monitoring should be incorporated into clinical care.

## Appendix

Multimedia Appendix 1 Technology Acceptance Questionnaire.

Multimedia Appendix 2 Analysis of data gaps.

## Abbreviations

aHAH: acute hospital at home
BP: blood pressure
ECG: electrocardiogram
EIP: electrical impedance pneumography
NEWS2: National Early Warning Score 2
NHS: National Health Service

## Data Availability

The datasets generated or analyzed during this study are available from the corresponding author on reasonable request.
