# Supplementary material for: Remote Vital Sign Monitoring in Acutely Unwell Hospital at Home Patients: Nonrandomized Feasibility Study
Source: JMIR Form Res. 2026 Sep 25;10:e84233. doi: 10.2196/84233 (PMC13653201; doi:10.2196/84233)
Supplement: Supplementary file 1 [file formative_v10i1e84233_app1.docx]

Participant Study ID:

CI: Prof Andrew Farmer

Research Team Contact: 01865 231448

# VIABLE – TECHNOLOGY ACCEPTANCE QUESTIONNAIRE

# Remote monitoring in virtual wards for acutely unwell patients being managed and treated on an ambulatory care pathway: feasibility study

The following information is to be collected from the participant verbally. Please read the following to the participant:

***I would like to ask you some questions about your experience using monitoring devices while you were being looked after by the Hospital at Home team. These were: a vital-sign chest patch which was a sticky patch on your chest; a pulse oximeter that went on your finger; a blood pressure monitor that wrapped around the top of your arm; and a thermometer you put in your ear.***

***I’m going to read you a series of statements about your experiences and ask you to rate how much you agree or disagree with each statement. Depending on your answer I may ask you to explain a bit about why you feel that way. I will be writing down your responses. There are six statements and you are welcome to ask me any questions you may have as we go through. Do you have any questions for me now?***

Answer any questions the participant may have before going through each of the statements below. Statements should be rated with the following scale:

| Completely disagree | Mostly disagree | Somewhat  disagree | Neutral | Somewhat agree | Mostly agree | Completely agree |
| --- | --- | --- | --- | --- | --- | --- |
| **1** | **2** | **3** | **4** | **5** | **6** | **7** |

**Questionnaire**

**If the participant disagrees with a statement please ask why they feel that way and record their response in the comments box.** Please add any comments the participant may have to the relavant comment box.

| **Statement** | **Rating (1 – 7)** |
| --- | --- |
| 1. Using the monitoring devices whilst I was unwell was easy for me |  |
| Comments: | |
| 2. It was easy to get the monitoring devices to do what I wanted them to do |  |
| Comments: | |
| 3. What I needed to do with the monitoring devices was clear and understandable |  |
| Comments: | |
| 4. It was easy for me to learn what I need to do with the monitoring devices |  |
| Comments: | |
| 5. I found the monitoring devices easy to use |  |
| Comments: | |
| 6. I felt that having the monitoring devices was useful for my health care |  |
| Comments: | |
