# Supplementary material for: Remote Vital Sign Monitoring in Acutely Unwell Hospital at Home Patients: Nonrandomized Feasibility Study
Source: JMIR Form Res. 2026 Sep 25;10:e84233. doi: 10.2196/84233 (PMC13653201; doi:10.2196/84233)
Supplement: Supplementary file 2 [file formative_v10i1e84233_app2.docx]

**Analysis of data gaps**

The mean proportion of missing data (passive monitoring – VitalPatch heart rate values) during the daytime (08:00–20:00) was 15% (SD 15%) with a median of 9% (IQR 1%–24%). During the nighttime (20:00–08:00), the mean proportion of missing data was 22% (SD 26%) with a median of 9% (IQR 2%–42%). The higher variability in the nighttime missingness may reflect participants not consistently keeping the tablet gateway device in close proximity overnight.

The average number of 1–4 hour gaps per participant was 4.48 (SD 6.69), with a median of 0 (IQR 0–5). The average number of gaps exceeding 4 hours was 1.52 (SD 2.14), with a median of 1 (IQR 0–2). The relatively small number of prolonged (>4 hour) gaps suggests that the system can generally support observation intervals comparable with the 4-hourly NEWS2 assessments used in hospital practice.

In Figure A1, a heatmap of the data availability and gaps per participant is plotted, ordered by missingness percentage. Visual inspection of the start of the gap times did not demonstrate a consistent tendency for gaps to occur at particular times of day. The proportion of missing observations did not demonstrate a clear hour-of-day pattern, suggesting that missingness was not associated with time of day.

The total recorded step count during monitoring is also displayed in Figure A1 alongside each participant to facilitate visual assessment of the relationship between participant activity and missing data. Given the relatively small sample size and the non-normal distribution of both variables (total recorded step count and proportion of missing physiological data), Spearman's rank correlation coefficient was used to assess the association between the two. Participant activity was moderately positively associated with missing data (Spearman's ρ = 0.63, p < 0.001), suggesting that more active participants experienced a greater proportion of temporary data loss. This finding is consistent with temporary loss of connectivity when participants moved beyond the range of the tablet device.

Figure S1 –Heatmap of data availability and gaps per participant, ordered by missingness percentage, along with total step count.

Generative artificial intelligence (ChatGPT Education – Oxford University License, GPT-5.5, OpenAI, 2026) was used to generate this Figure.
